# Degradation of Dyes Catalyzed by Aminophenyl-Substituted Mn-Porphyrin Immobilized on Chloropropyl Silica Gel and Evaluation of Phytotoxicity

**DOI:** 10.1021/acsomega.4c02132

**Published:** 2024-06-25

**Authors:** Igor Muniz de Oliveira, João Victor Docílio Pereira, Everton Carlos da Silva Pereira, Micaelle Silva de Souza, Márcia Luciana Cazetta, Claudiano Carneiro da Cruz Neto, Victor Mancir da Silva Santana, Victor Hugo Araújo Pinto, Júlio Santos Rebouças, Dayse Carvalho da Silva Martins, Gilson DeFreitas-Silva, Denilson Santos Costa, Vinicius Santos da Silva

**Affiliations:** † Centro de Formação de Professores, Universidade Federal do Recôncavo da Bahia, 45300-000. Amargosa, Bahia, Brazil; ‡ Centro de Ciências Exatas e Tecnológicas—Universidade Federal do Recôncavo da Bahia, 44380-000. Cruz das Almas, Bahia, Brazil; § Centro de Ciências Agrárias Ambientais e Biológicas—Universidade Federal do Recôncavo da Bahia, 44380-000. Cruz das Almas, Bahia, Brazil; ∥ Instituto de Física, Universidade Federal da Bahia, 40210-340 Salvador, Bahia, Brazil; ⊥ Departamento de Química, CCEN, Universidade Federal da Paraíba, 58033-455 João Pessoa, Paraíba, Brazil; # Departamento de Química, Instituto de Ciências Exatas, Universidade Federal de Minas Gerais, 31270-901 Belo Horizonte, Minas Gerais, Brazil; ¶ Instituto de Química, Universidade Federal da Bahia, 40170-115 Salvador, Bahia, Brazil

## Abstract

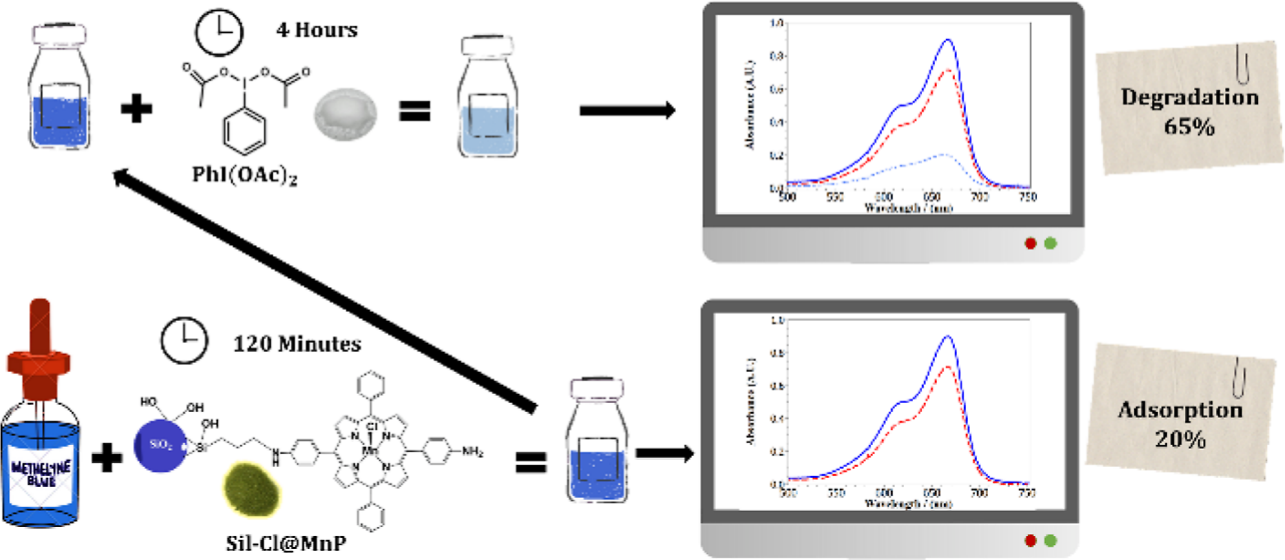

A heterogenized Mn(III) porphyrin-based catalyst was prepared for dye degradation. The new Mn(III) complex of 5,15-bis(4-aminophenyl)-10,20-diphenylporphyrin was immobilized, via covalent bond, in chloropropyl silica gel, generating the material (Sil-Cl@MnP) with a loading of 23 μmol manganese porphyrin (MnP) per gram of Sil-Cl. This material was used as a catalyst in degradation reactions of model dyes, a cationic dye [methylene blue (MB)] and an anionic dye (reactive red 120, RR120), using PhI(OAc)_2_ and H_2_O_2_ as oxidants. The oxidation reactions were carried out after the dye reached adsorption/desorption equilibrium with the catalytic material, with a much higher percentage of adsorption being observed for the cationic MB dye (20%) than for the anionic RR120 dye (3%), which may be associated with electrostatic attraction or repulsion effects, respectively, with the negatively charged surface of the silica (zeta potential measurement for Sil-Cl@MnP, ζ = −19.2 mV). In general, there was a higher degradation percentage for MB than for RR120, probably because the size and charge of RR120 would hinder its approach to the MnP active species on the silica surface. With respect to the oxidant, the PhI(OAc)_2_-based systems showed a higher degradation percentage than those of H_2_O_2_. It was observed that the increase in the oxidant concentration promoted a significant increase in the degradation of MB, with a degradation of approximately 65%. The efficiency of the catalyst was also evaluated after successive additions of the oxidant every 2 h, and it can be seen that the catalyst had no loss of efficiency, with a degradation percentage greater than 80% being observed after 8 h of reaction. The phytotoxicity of the products formed in the system was evaluated in a 1:23.5:188 molar ratio Sil-Cl@MnP: MB:PhI(OAc)_2_ was used. In these studies, phytotoxicity was found for the germination of lettuce seeds when the original solution was used without dilution; however, when diluted (10% V/V), the results were close to the positive and negative controls. Thus, the material obtained proved to be a potential candidate for application in the degradation reactions of environmental pollutants.

## 1 Introduction

The large-scale use of dyes by industries in different sectors, such as textiles, leather, paints, food, printing, and paper manufacturing, associated with the lack of waste treatment and inadequate disposal, has been one of the main sources of environmental pollution.^1^ The main environmental problems associated with dyes occur when they are dumped into rivers, lakes, and effluents, as they cause changes in the aesthetics of the water, due to changes in color, which interrupts photosynthesis in aquatic plants, initiating the eutrophication process.^2^

**Figure 1:**
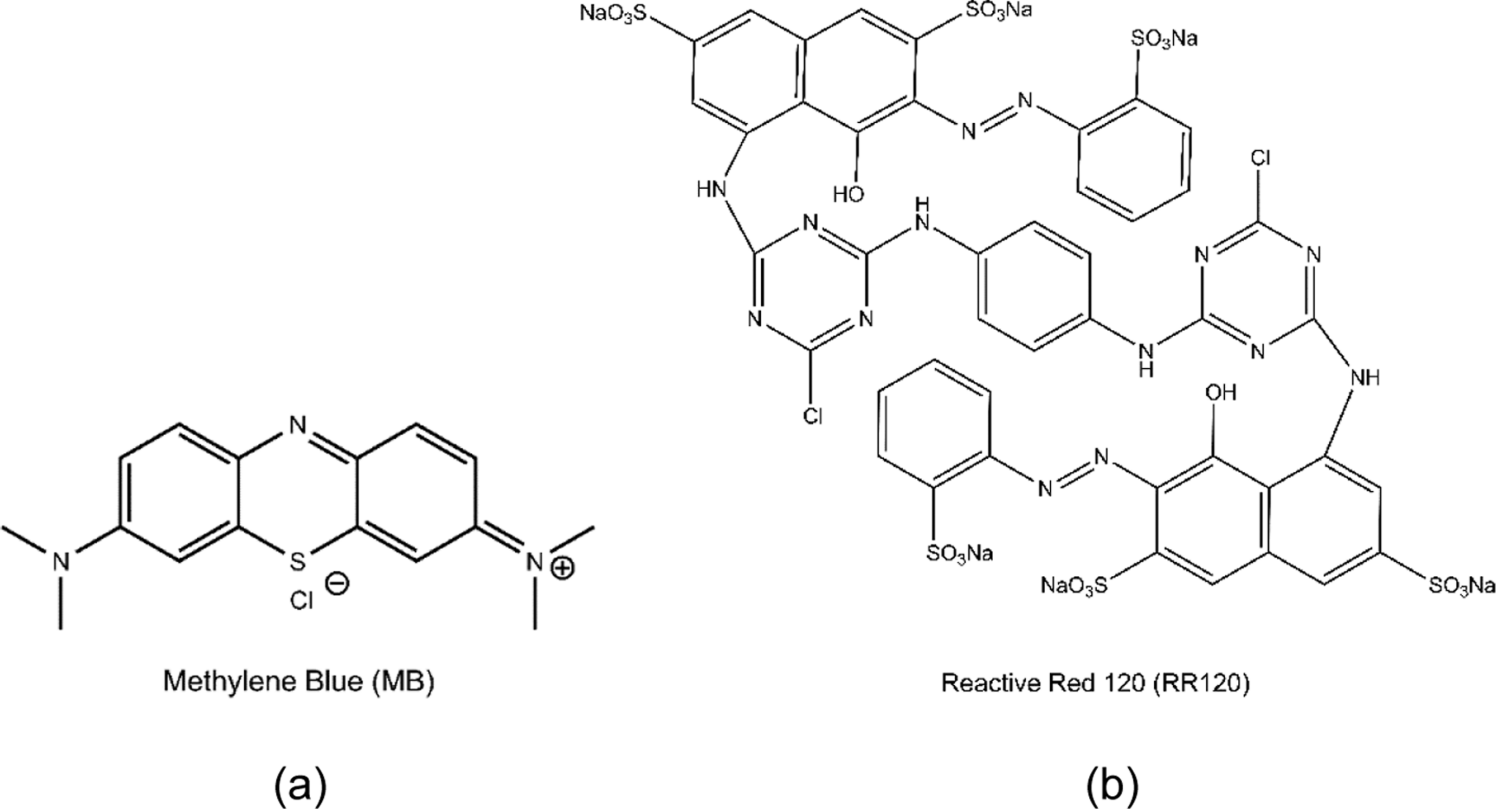
Chemical structure of (a) MB and (b) reactive red 120.

Methylene blue [MB; 3,7-bis(dimethylamino)-5-phenothiazinium chloride; Figure 1], a cationic organic dye used in several areas of science, including medicine, is widely used as a model in degradation reactions of organic pollutants. This is due to its high chemical stability and high adsorption on solid supports.^3^

The dyes that present in their structure the azo group (–N=N–), which unites two identical symmetric and/or nonsymmetric groups or non-azo alkyl or aryl radicals, since they are toxic, carcinogenic, mutagenic, resistant to conventional treatment processes and are not biodegradable, such as the anionic dye reactive red 120,^4^Figure 1.

Faced with the serious problems caused by these pollutants, different techniques have been proposed by the scientific community to degrade these contaminants, such as photolysis of H_2_O_2_ by UV irradiation, Fenton-type reactions, photo-Fenton, heterogeneous photocatalysis,^5^ and biomimetic catalysis,^6,7^ which consists of designing high-performance catalysts using biological systems as a source of inspiration.^8^

Among the most common biomimetic models are those of cytochromes P450 (CYP), which are based on the development of metalloporphyrin catalysts and use in different transformations that include fine chemistry and the degradation of different classes of pollutants.^8−10^

The use of metalloporphyrins as CYP biomimetic catalysts has been carried out since the 1980s.^11^ Since then, different strategies have been implemented by the scientific community to increase the efficiency of these catalytic systems, such as the introduction of functional groups on the periphery of the porphyrin macrocycle,^8^ the use of additives such as imidazole and water^12,13^ and immobilization on different inorganic and/or organic supports.^14^

The immobilization of metalloporphyrins on chemical supports and their use in heterogeneous catalytic processes appears as an alternative to overcome the limitations inherent to homogeneous systems.^15−21^ A variety of supports, both organic and inorganic, have been used to anchor (immobilize and support) metalloporphyrins. Inorganic supports have an advantage over organic polymers because they are more resistant to oxidative processes, more robust, more rigid, thermally stable, and resistant to organic solvents, and the immobilization reaction conditions are simpler.^16,18,21−26^

Among the most studied surfaces as supports, silica gel stands out (in natura, chemically modified, prepared via sol–gel processes in the presence or absence of the catalyst, of porous/nonporous structures, and so on).^25^ Silica gel is formed by silicon tetrahedra linked to four oxygen atoms, essentially formed by Si–O–Si (siloxane bridges) and Si–OH (silanol group) bonds, with silanols being mostly found on the surface in a disordered distribution.^27^ The presence of silanol groups confers a negative charge density on the silica surface and allows surface modification, for example, by the addition of spacers, such as alkyl groups, which allows metalloporphyrins to be associated with these spacers, giving rise to more catalytically efficient and robust systems.^25^

Manganese porphyrins with one,^12^ two,^28^ or four^29^ 4-aminophenyl substituent in the para positions of meso-aryl groups have been used as biomimetic catalysts in oxidation reactions of organic substrates in a homogeneous medium, presenting high efficiency in these systems, which makes this class of compounds extremely interesting to be associated with inorganic supports. Although there are many studies involving metalloporphyrins immobilized on silica (via electrostatic interactions or covalent bonding),^19,20,25,30−32^ there are few studies that describe the use of metalloporphyrins with the aminophenyl substituent immobilized via covalent bonding on inorganic/organic supports.^33,34^

We hypothesized that obtaining a material based on the covalent immobilization of a new nonsymmetrical 4-diaminophenyl MnP on silica gel functionalized with chloropropyl groups may generate a robust material of high efficiency for use as a catalyst in dye degradation reactions. The use of an amorphous silica gel as an inert support has been very interesting because it is cheap, ordinary, and widely used for column chromatography. In a previous report, Pinto and co-workers^32^ studied SiO_2_ containing covalently bound cationic manganese porphyrins. They verified that SiO_2_ (with or without Cl-groups) was both more efficient and equally selective for cyclohexane oxidation than SBA-15-based catalysts, but for *n*-heptane oxidations, these same materials showed different chemoselectivity. The covalent immobilization of these catalysts on Sil-Cl, through Cl-groups, allowed recycling studies due to the low leaching/destruction of the supported Mn porphyrins.

In pollutant degradation systems catalyzed by metalloporphyrins, the oxidants play an important role, as they can drive reactions through radical mechanisms, as in the case of H_2_O_2_ and OXONE, or via nonradical (oxygen transfer) mechanisms, as in the case of PhIO and PhI(OAc)_2_. Although not environmentally friendly, these hypervalent iodine-based oxidants are good models to evaluate the role of the catalyst in reactions.^35^

**Figure 2:**
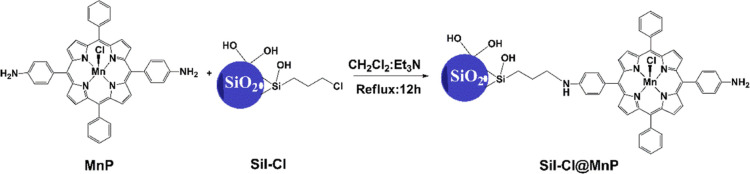
Structural representation of catalytic material obtention.

Thus, this work aimed to immobilize a new Mn(III)-porphyrin that presents two 4-aminophenyl substituents in a *trans* fashion with respect to the macrocycle (MnP, Figure 2), to silica gel functionalized with chloropropyl groups (Sil-Cl) and used the resulting material (Sil-Cl@MnP) as a biomimetic catalyst in degradation reactions of two model dyes: a cationic one (MB) and an anionic one (reactive red 120) using H_2_O_2_ and PhI(OAc)_2_ as oxidants. To the best of our knowledge, there is no study presenting a nonsymmetrical diamino-porphyrin (in a trans fashion with respect to the macrocycle) covalently attached to silica support, and consequently, no catalytic studies are using this type of material. Additionally, the phytotoxicity of the products formed in the solution resulting from the catalytic treatment versus that of the original dye solution was evaluated.

## 2 Results and Discussion

### 2.1 Catalyst Preparation

**Figure 3:**
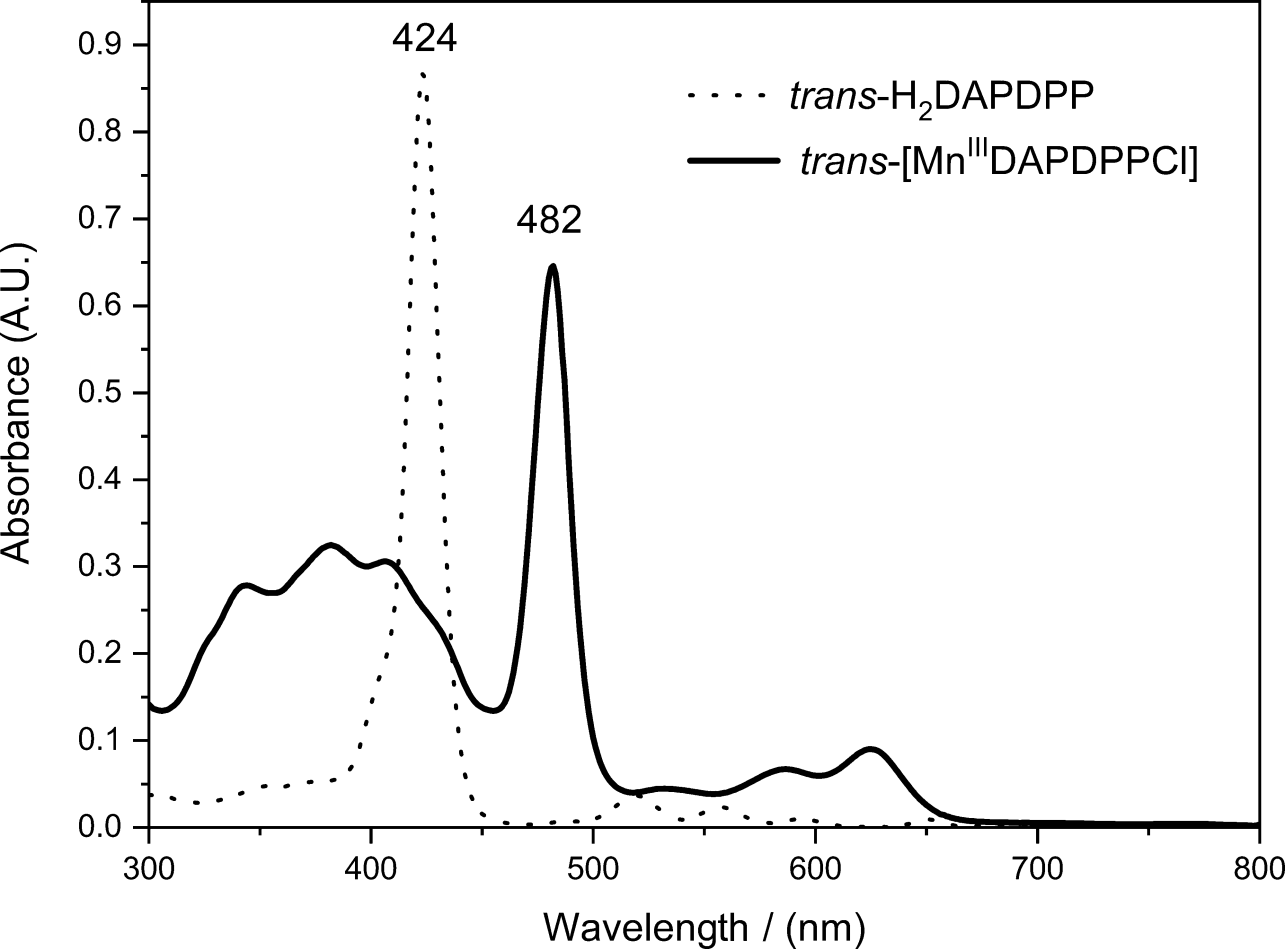
UV–VIS spectra of the starting material *trans*-H_2_DAPDPP and the new complex *trans*-[Mn^III^DAPDPPCl] in dichloromethane at concentrations of 3.11 and 0.15 μmol L^–1^, respectively.

The new MnP (*trans*-[Mn^III^DAPDPPCl]) was obtained via metalation of the corresponding free-base porphyrin ligand *trans*-H_2_DAPDPP^12^ using the chloroform/methanol method^36^ and initially purified by liquid–liquid extraction to remove the excess of Mn(II) salt. Next, the resulting material in the organic phase was purified by column chromatography, through which it was possible to separate the unreacted H_2_P and the MnP of interest. The porphyrin compound was characterized by UV–vis spectroscopy (Figure 3), infrared spectroscopy [Fourier transform infrared (FTIR)], and high-resolution mass spectrometry, as shown in the Supporting Information.

The spectrum of the free base porphyrin is in accordance with Gouterman’s four orbital model, with the Soret band at ∼420 nm and four Q bands of lower intensity between 500 and 650 nm, even though the porphyrin does not belong formally to the point group *D*_4*h*_.^37^ This is because the substituents in the para positions of the mesoaryl groups do not significantly influence the energies of the frontier orbitals. The UV–vis spectrum for the MnP complex is characteristic of a type d hyperporphyrin, in which there is a bathochromic shift of the Soret band, in relation to H_2_P, the appearance of a metal–ligand charge transfer (MLCT) band (centered at 381 nm), and a decrease in the number of Q bands. MLCT bands occur due to the migration of charges from molecular orbitals higher energy occupied of the metal to the lower energy unoccupied molecular orbitals of the porphyrin.^38^

The samples of unmodified silica gel (SiO_2_) and the functionalized chloropropyl silica gel (Sil-Cl) were derived from our previous study^32^ being, thoroughly characterized by elemental analysis (C and Cl), FTIR, thermogravimetric analysis-DTA, ^13^C NMR (Sil-Cl), ^29^Si NMR (Sil-Cl), specific surface area (Brunauer–Emmett–Teller method), adsorption–desorption isotherms (Sil-Cl), pore size distribution (BJH method, Sil-Cl), scanning electron microscopy (SEM), and transmission electron microscopy, as reported elsewhere.^32^ The immobilization of MnP on the functionalized silica gel (Figure 2) occurred via covalent bonding through a nucleophilic substitution of chlorine by the aminophenyl group, generating hydrochloric acid (HCl) as a byproduct. To neutralize the hydrochloric acid formed in situ, avoiding both the demetalation of MnP and the protonation of the remaining aminophenyl group, triethylamine, a sterically hindered Brønsted-Lowry base, was added in large excess. This functionalization of the inorganic support showed a yield of 38% and a loading of 23 μmol of MnP per gram of Sil-Cl. This loading value is approximately 6-fold higher than that reported for the immobilizing of Mn(III) *N*-pyridylporphyrins series on Sil-Cl,^32^ although the immobilization yields in the pyridyl cases were close to 100%. This difference is possibly due to the lower molar ratio used in the literature (4 μmol g^–1^), whereas in this work we started the immobilization reaction with a ratio close to 60 μmol g^–1^, in order to obtain a material with high loading. Although the chloropropyl-functionalization of silica is on the order of 930 μmol g^–1^, we infer that there was a saturation of MnP on Sil-Cl, as MnP is able to (i) bind to Sil-Cl through two amino groups and (ii) lead to steric hindrance on the surface due to the size of the porphyrin macrocycle.

**Figure 4:**
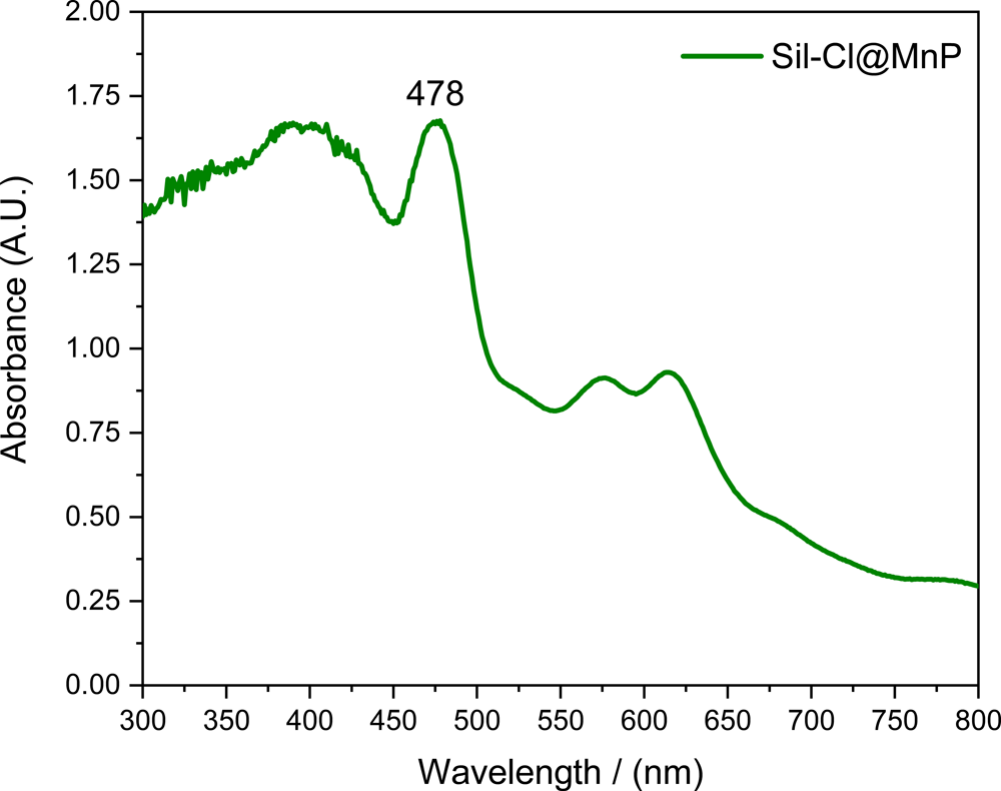
Diffuse reflectance UV/vis spectra of Sil-Cl@MnP.

The immobilization of MnP to silica can be confirmed by the intense greenish color of the silica after the reaction and workup procedures. Furthermore, the analysis of the DRS-UV/vis spectrum of the Sil-Cl@MnP (Figure 4) revealed that there were no significant changes in the porphyrin macrocycle or the metal center ion, as it was possible to observe the charge transfer bands around 380 nm, the Soret band (∼478 nm) and the two Q bands (between 500 and 650 nm), denoting the presence of MnP with the metal ion with oxidation state 3+ on the silica surface.^38^

Immobilization by covalent bonding via the periphery of the porphyrin macrocycle keeps the Mn-porphyrin away from the support due to the organic spacer chain, therefore minimizing the steric and polarizing effects of the surface,^39^ differently from what would occur if the immobilization was via the porphyrin metal center.

**Figure 5:**
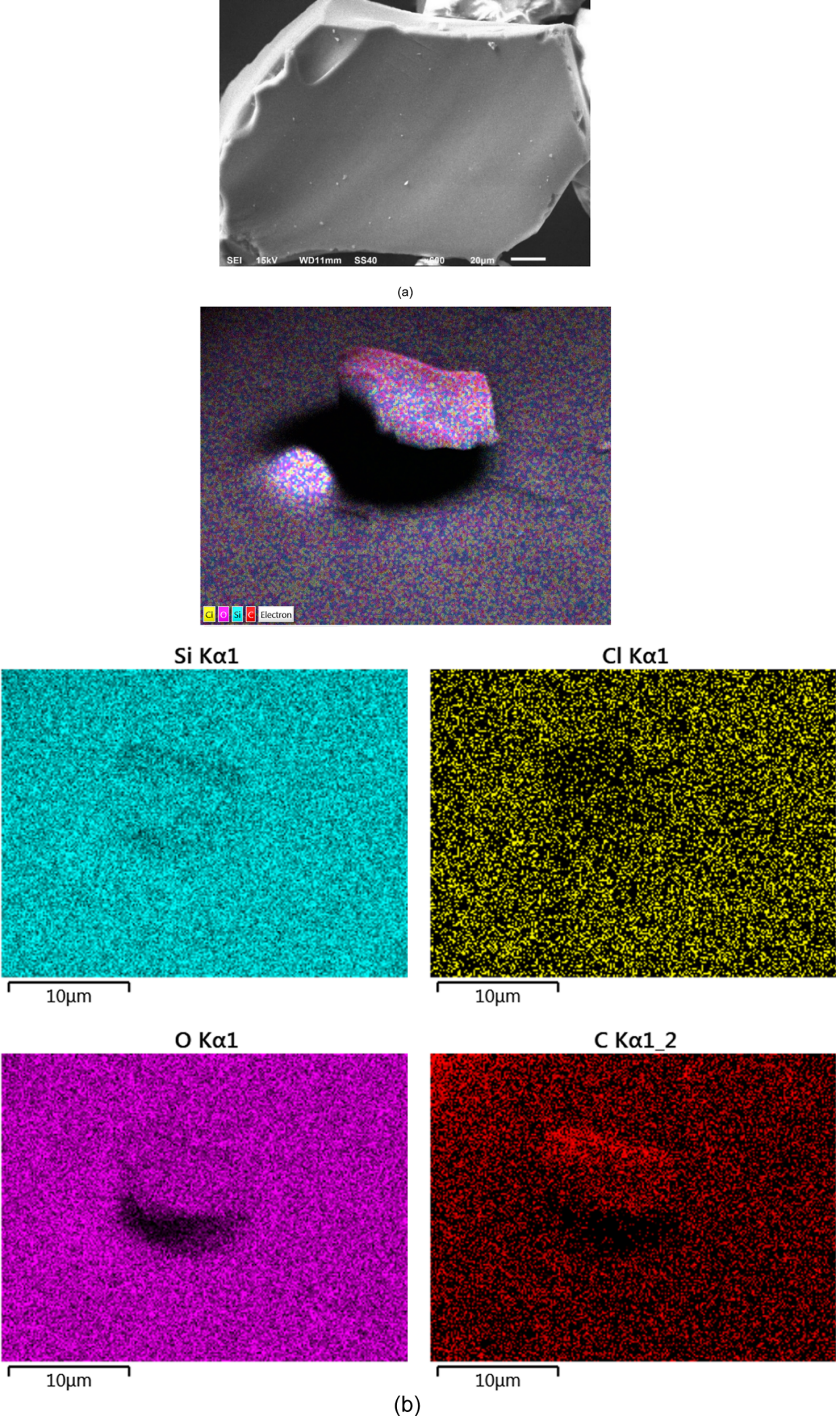
(a) Scanning electron micrograph of the catalytic material Sil-Cl@MnP and (b) micrograph with the distribution of chemical elements present in Sil-Cl@MnP. The colors represent different chemical elements: red, carbon; blue, silicon; pink, oxygen; and yellow, chlorine.

The material (Sil-Cl@MnP) was characterized by FTIR (Supporting Information). However, in this spectrum, it is not possible to highlight vibrational modes associated with MnP because the concentration of MnPs in silica is very low and does not allow its detection. In order to investigate the morphology and carry out a mapping of the chemical elements present on the surface, the catalyst Sil-Cl@MnP was analyzed by SEM and SEM-EDS (Figure 5).

When comparing the micrograph of the starting functionalized silica gel^32^ to the Sil-Cl@MnP, it is possible to propose that the immobilization of MnP to Sil-Cl did not significantly alter the morphology of the silica. Analyzing Figure 5b, it is possible to identify the constituent elements of Sil-Cl distributed uniformly in the sample; however, the MnP elements such as Mn and N could not be detected, due to the relatively low concentration of these chemical elements in relation to the other constituent elements of the sample. Other characterization techniques, such as thermal analysis, and elemental analysis, are not suitable for confirming the form of immobilization of MnP in Sil-Cl. This limitation arises from the fact that the percentage of MnP in silica is less than 2%, which is a common constraint observed in studies involving the heterogenization of MnP.^32,40^

### 2.2 Dye Degradation Catalyzed by Sil-Cl@MnP

The degradation reactions of MB and RR120 dyes were conducted initially by evaluating the nature of the oxidant [H_2_O_2_ and PhI(OAc)_2_] and the molar ratio catalyst:dye:oxidant. Control reactions were also carried out in the absence of the oxidant, in the absence of the catalyst, and/or in the absence of the catalyst and oxidant keeping the other overall conditions and reactants the same.

When evaluating the stability of the MB and RR120 solutions, it was found that they were stable throughout the reaction time (4 h), in the absence of catalyst and oxidant, with no significant variation observed in the absorbance of these dye solutions (entries 1 and 11, Table 1), indicating their stability against molecular oxygen.

**Table 1:** Degradation of Dyes by PhI(OAc)_2_ or H_2_O_2_ Catalyzed by Sil-Cl@MnP

entry	catalyst (Cat)	oxidant (Ox)	dye (D)	molar ratio (Cat:Ox^a^)^b^	% degradation^c^
1			MB		0
2		H_2_O_2_	MB	(0:2950)	3
3	Sil-Cl@MnP	H_2_O_2_	MB	(1:2950)	8
4		H_2_O_2_	MB	(0:5900)	5
5	Sil-Cl@MnP	H_2_O_2_	MB	(1:5900)	8
6		PhI(OAc)_2_	MB	(0:23.5)	7
7	MnP	PhI(OAc)_2_	MB	(0:23.5)	5
8	Sil-Cl@MnP	PhI(OAc)_2_	MB	(1:23.5)	20
9		PhI(OAc)_2_	MB	(0:47)	10
10	Sil-Cl@MnP	PhI(OAc)_2_	MB	(1:47)	30
11			RR120		0
12		PhI(OAc)_2_	RR120	(0:23.5)	7
13	Sil-Cl@MnP	PhI(OAc)_2_	RR120	(1:23.5)	13
14		PhI(OAc)_2_	RR120	(0:47)	19
15	Sil-Cl@MnP	PhI(OAc)_2_	RR120	(1:47)	28

a D: dye: the same molar ratio was maintained in all systems, with a 23.5-fold molar excess in relation to the catalyst.

b Cat: catalyst, D: dye, Ox: oxidant.

c The degradation reactions were carried out in distilled water, under mild conditions *T* = 25 °C, atmospheric pressure, and magnetic stirring, in the absence of light, for 4 h.

**Figure 6:**
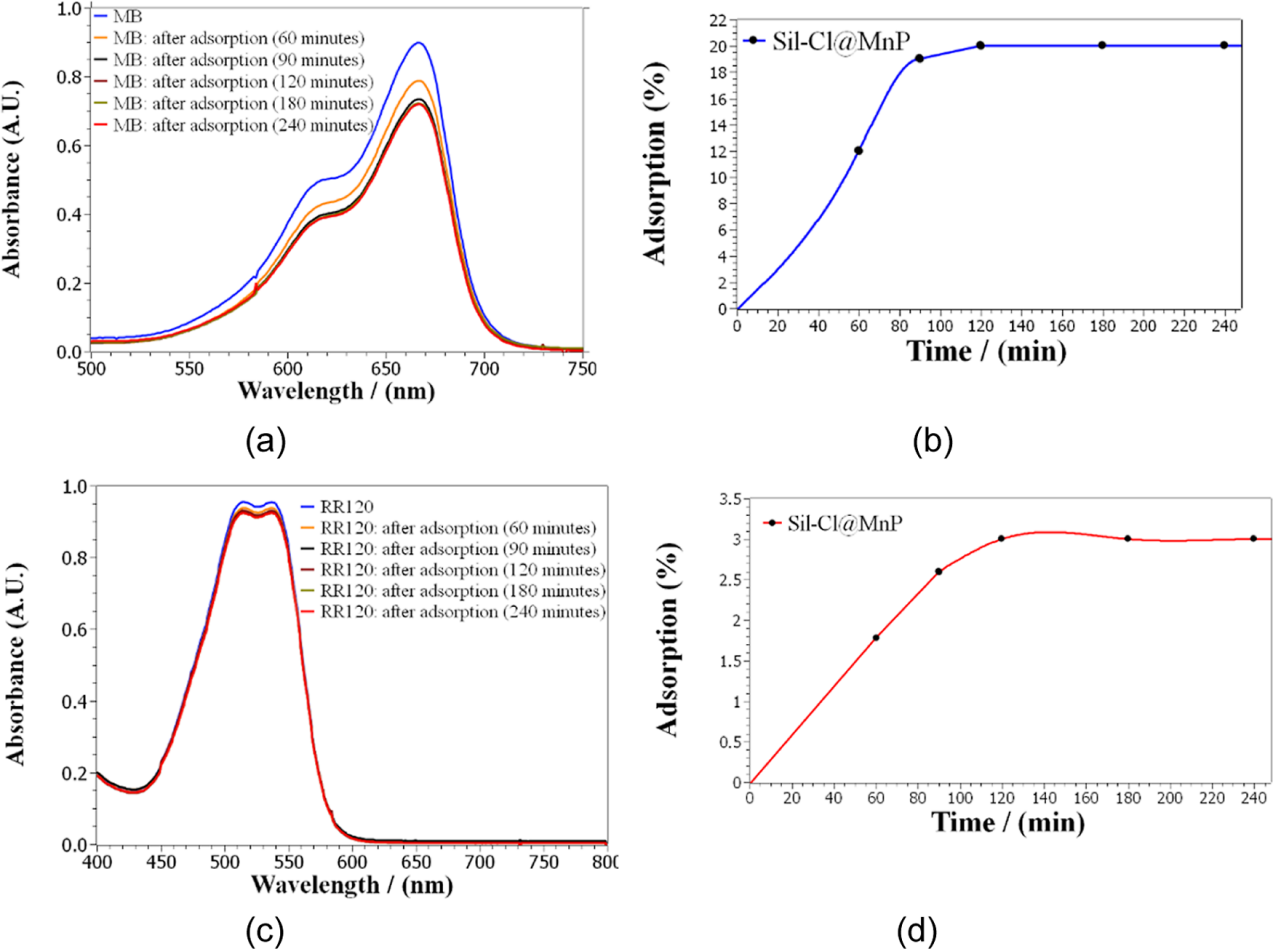
(a) UV–vis spectra MB in the presence of Sil-Cl@MnP in distilled water. (b) MB adsorption percentage on Sil-Cl@MnP as a function of time. (c) UV–vis spectra RR120 in the presence of Sil-Cl@MnP in distilled water. (d) RR120 adsorption percentage on Sil-Cl@MnP as a function of the time.

Tests to evaluate the percentage of adsorption of dyes were also carried out, in which there was a decrease of ∼20% in MB concentration (Figure 6) and ∼3% for reactive red in 90 min, remaining constant for up to 24 h, the time at which the last analysis was carried out. This large difference in the degree of adsorption between MB and RR120 may be associated with their respective cationic and negative nature and the negative charge of the silica surface (Sil-Cl@MnP, ζ = −19.2 mV), which favors the interaction with the cationic MB dye as opposed to the anionic RR120 dye.

The stability of the dye solutions after 90 min for up to 24 h (Figure 6B for MB) indicates that even in the presence of the catalyst Sil-Cl@MnP molecular oxygen did not promote the oxidation of the dyes.

MB degradation reactions were initially conducted using H_2_O_2_ as an oxidant. This oxidant yields water as a byproduct, being in accordance with the principles of green chemistry.^30^ The molar ratio established for these systems was based on the work described by Ucoski et al. (2015),^41^ in which the authors used Mn-porphyrins on different supports for the degradation of brilliant green. In catalytic systems involving Mn-porphyrins and peroxides, the substrate oxidation process can occur through two mechanisms: (a) heterolytic cleavage of the O–O bond, which will produce the high-valence active species (Mn^v^=O) and (b) homolytic cleavage of the O–O bond, which will produce the least reactive species (Mn^IV^–OH).^42^

In the control reaction without the catalyst (entry 2, Table 1), a 3% degradation of the dye was observed, while in the system with the catalyst (entry 3, Table 1) in the molar ratio (1:2950:20) there was a slight increase in dye degradation (8%). When the amount of oxidant was doubled (entries 4 and 5, Table 1), there was a slight increase in MB degradation in the system without the catalyst, and no changes were observed in the system with the catalyst. These results are in agreement with the results described by Almeida Lage et al. (2019),^43^ that in the degradation of atrazine by Mn-porphyrins, a degradation of only ca. 4% was achieved when using the H_2_O_2_ as oxidant. The authors suggested that the degradation occurs predominantly via the (Mn^IV^–OH) species, which is less reactive. Furthermore, reactions involving hydrogen peroxide are favored in a basic medium, pH between 10 and 12, which promotes the formation of the radical species superoxide anion and hydroxyl radical.^30^ However, in this work, we carry out the reactions at native pH, that is, we do not adjust the pH of reaction mixture, which may explain the lower results observed in our work, in relation to the work developed by Zucca et al. (2012), that used a Mn-porphyrin immobilized in silica.^44^ Furthermore, in the work developed by Zucca et al. (2012), the pronounced increase in dye degradation can be justified by the form of immobilization via imidazole, which may act as a cocatalyst favoring the cleavage of the oxidant.^13,28^

Based on the low degradation of MB observed with hydrogen peroxide, an alternate, hypervalent iodine oxidant, PhI(OAc)_2_, was also investigated. This oxidant is the precursor of PhIO, a classic oxidant used since the first catalytic studies with synthetic metalloporphyrins. As reported by our group and other researchers, PhI(OAc)_2_ presents similar or even superior results to those of PhIO-based oxidations.^28,45^ However, there is still no consensus about the mechanism by which the formation of the high-valence active species occurs. (Mn^V^(O)P) with this oxidant, although there are already some kinetic studies that indicate some possibilities.^35,46−48^ In and collaborators, for example, suggest that the hydrolysis of PhI(OAc)_2_ in the presence of water occurs, generating PhIO in situ.^49^ Additionally, PhI(OAc)_2_ eliminates the synthesis and isolation of PhIO, is stable at room temperature, soluble in most organic solvents, and has lower toxicity than PhIO.^50,51^

In the degradation reactions of MB with PhI(OAc)_2_ and without catalyst (entry 6, Table 1) a degradation percentage of 7% was found, the system contained MnP (without immobilized) and PhI(OAc)_2_ the degradation was 5% (entry 7, Table 1), while in the presence of the immobilized catalyst (Sil-Cl@MnP), the degradation was 20% (entry 8, Table 1), indicating that PhI(OAc)_2_ in the presence of Sil-Cl@MnP generated an active species of high valence, responsible for the substrate oxidation, also denoting the importance of the catalyst for the activation of the oxidant in these systems. By doubling the amount of oxidant (entries 9 and 10, Table 1), an increase in the catalytic degradation of MB (30%) with respect to the corresponding control reaction (10%) was observed, indicating the potential of the catalyst in this type of reaction.

When evaluating the degradation of reactive red 120, it was found that in the absence of a catalyst, the oxidant PhI(OAc)_2_ (entry 12, Table 1) led to the same percentage of degradation as in MB dye (7%). In the presence of catalyst and PhI(OAc)_2_, the degradation of RR120 practically doubled (13%), but was still lower than the one observed with MB, which may be related to (i) the greater steric hindrance of the azo chromophore in RR120 with respect to MB; and (ii) the unfavorable electrostatics between the anionic RR120 dye and the negative silica surface, which makes it difficult for RR120 to approach the catalytically active species. When the amount of oxidant was doubled, there was a significant increase (19%) in the degradation of RR120 in the system without the catalyst (oxidant only) and an increase to 28% of degradation for the system with the catalyst + oxidant (entries 14 and 15, Table 1). To the best of our knowledge, this is the first work that describes the oxidation of reactive red 120 using metalloporphyrins. Although the degradation yields do not indicate high degradation of this dye, it is important to note that adjustments to the system may eventually lead to more effective degradation, when using, for example, another immobilization support, preferably with positive charge surface.

When the MB and RR120 systems were compared, MB showed higher stability against the oxidant alone, but this dye was also more sensitive to Sil-Cl@MnP-based oxidative catalysis. Given the best results obtained with MB, it was decided to expand studies with this dye, evaluating the increase in the molar ratio of the oxidant substance and successive additions of the oxidant.

#### 2.2.1 Assessment of the Molar Ratio of Catalyst/Oxidant

**Figure 7:**
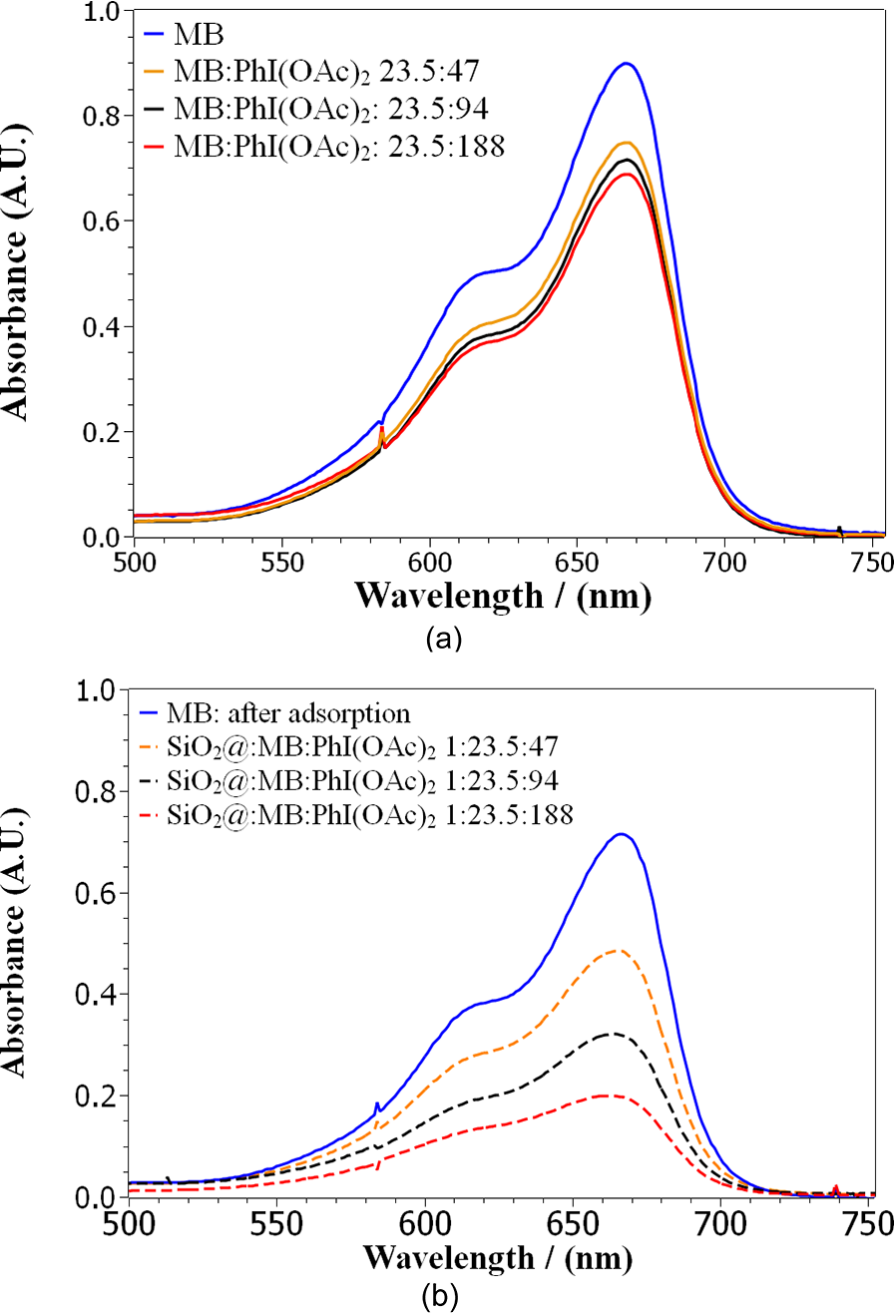
(a) UV–vis spectra MB in the exclusive presence of PhI(OAc)_2_ in 4 h using the ratio of quantity of catalyst/dye/oxidant substance (0:23.5:47), (0:23.5:94), and (0:23.5:188). (b) UV–vis spectra MB in the presence of Sil-Cl@MnP:PhI(OAc)_2_ in 4 h using the ratio of amount of substance catalyst:dye:oxidant (1:23.5:47), (1:23.5:94), and (1:23.5:188); the reactions were carried out in distilled water under mild conditions *T* = 25 °C, atmospheric pressure, and magnetic stirring in the absence of light.

**Figure 8:**
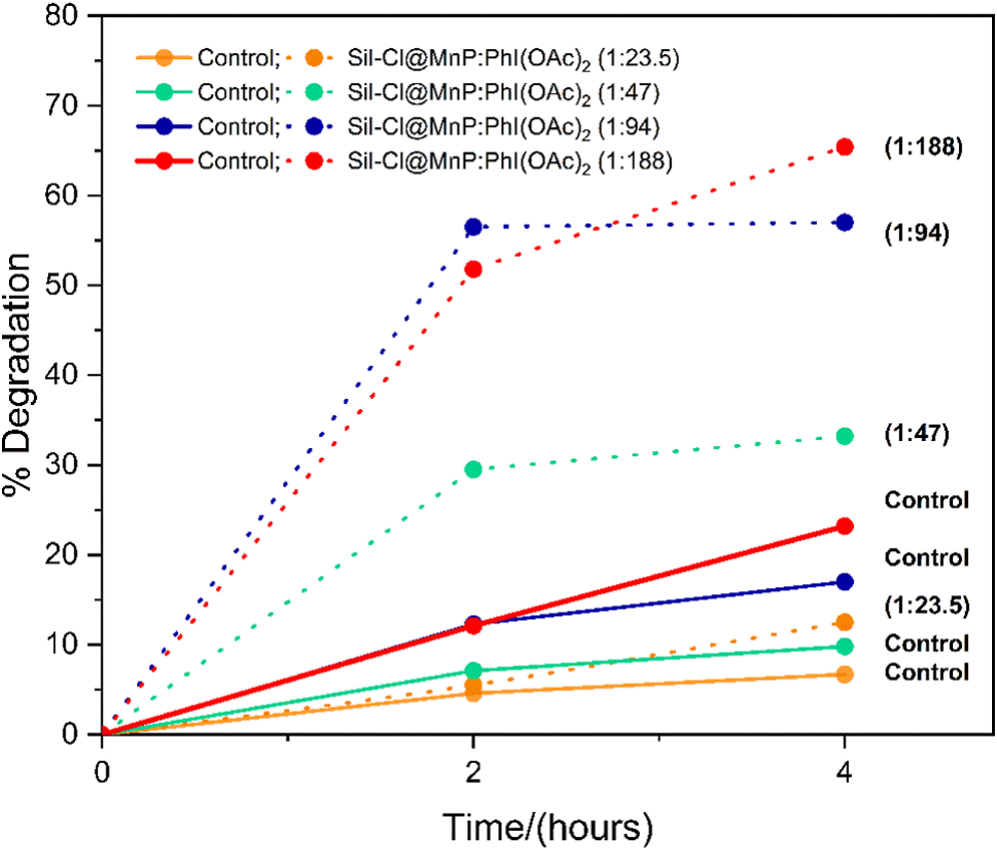
Percentage of MB degradation per PhI(OAc)_2_ catalyst by Sil-Cl@MnP in different relationships in the quantity of substance. Reactions were carried out in an aqueous solution, under magnetic stirring (temperature of 25 °C and atmospheric pressure).

For MB degradation reactions using PhI(OAc)_2_ as oxidant, the following relationships in quantity of dispersed substance were evaluated: oxidant (23.5:23.5), (23.5:47), (23.5:94), and (23.5:188) Figure 7. In control systems, that is, without the catalyst, the increase in the concentration of the oxidant promotes an increase in the degradation of the dye. The same behavior was verified for the systems in the presence of the catalyst Sil-Cl@MnP; however, the increase in MB degradation occurred to a much greater extent when compared to systems without the catalyst (Figures 7 and 8). In systems with the catalyst, there was also a very steep slope in the MB degradation curve in the first 2 h of reaction with only a slight increase thereafter. This slight increase may be related to the consumption of the remaining oxidant that was not consumed in the first 2 h of the reaction. The 1:94 ratio system, however, presented the highest degradation within 2 h with no further increase thereafter. It is worth noting that with the 23.5:188 ratio, the aqueous system may have reached saturation of PhI(OAc)_2_, given that a white precipitate associated with the oxidant was observed at the beginning of the reaction. However, after 4 h of reaction, the white precipitate was no longer verified, indicating that it was being consumed over time, justifying the increase in the percentage of MB degradation. A further assessment of the system with a molar ratio (23.5:188) was carried out by extending the reaction time to 8 and 24 h, but no further degradation was noted, indicating that the oxidant was all consumed within 4 h of reaction.

#### 2.2.2 Evaluation of Successive Oxidant Additions

**Figure 9:**
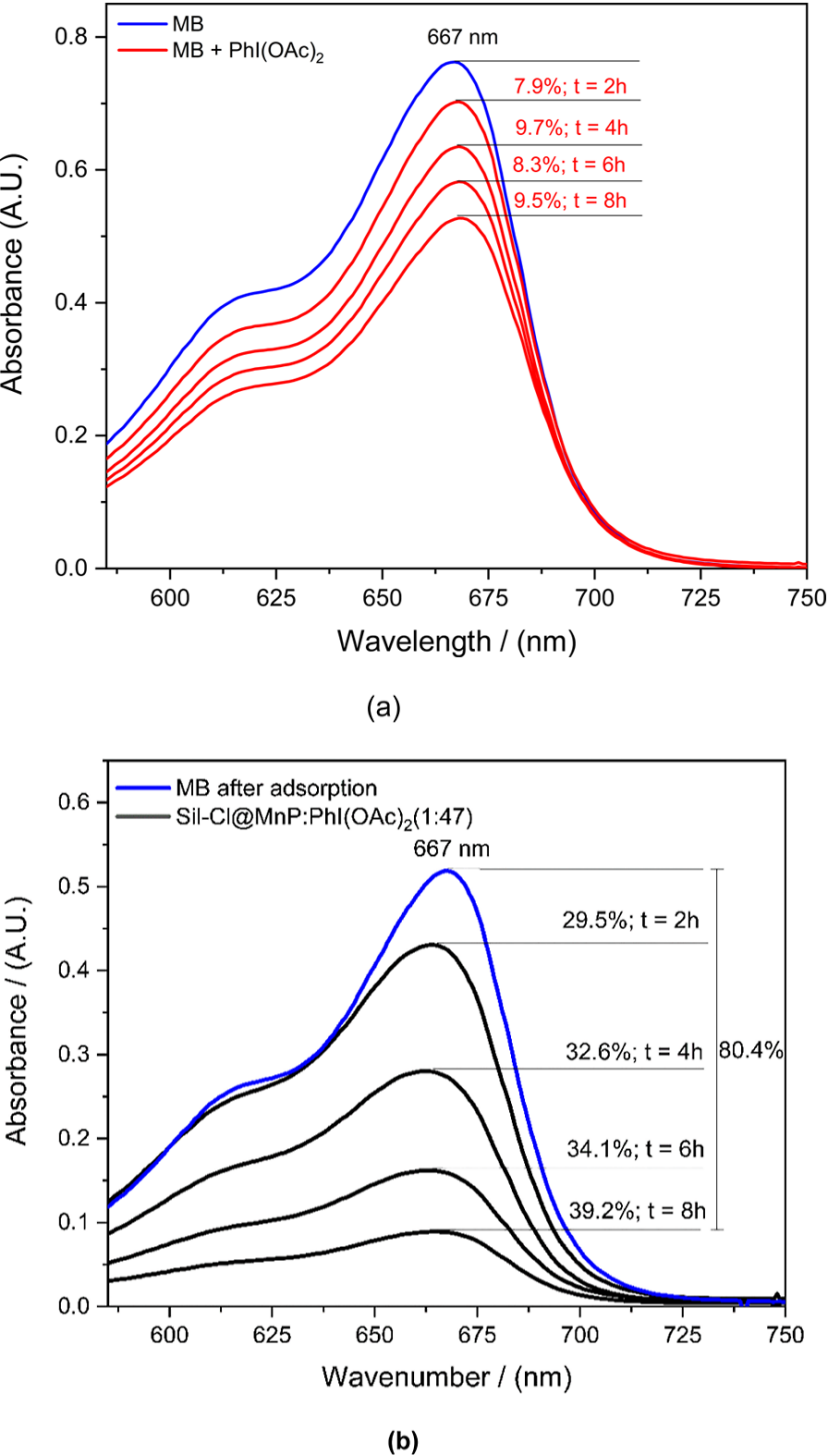
(a) UV–vis spectra of MB degradation reactions by PhI(OAc)_2_ using the following molar ratio MB:PhI(OAc)_2_ (23.5:47); (b) UV–vis spectra of MB degradation reactions by the PhI(OAc)_2_ catalyst by Sil-Cl@MnP using the following molar ratio Sil-Cl@MnP: MB:PhI(OAc)_2_ (1:23.5:47), with successive additions of oxidant every 2 h. To determine the 80% degradation, the absorbance of the MB solution after adsorption and the absorbance of this solution after 8 h.

For the system that presented the best result, molar ratio catalyst:oxidant (1:188), the oxidant is not fully soluble under these conditions, as discussed above. Thus, in an attempt to optimize this system, the oxidant was added in a partitioned manner. In order to account for the molar ratio catalyst:oxidant (1:188), a total of four successive additions of 47 equiv of PhI(OAc)_2_ every 2 h (i.e., at times 0, 2, 4, and 6 h), with sampling for MB degradation analyses at 0, 2, 4, 6, and 8 h, were carried out (Figure 9). The corresponding control reactions were also carried out under the same conditions, but in the absence of the catalyst.

In the control systems without the catalyst, there was a degradation of MB of ∼9% in each cycle, showing a constant degradation rate of the dye. In the catalytic systems, the degradation was 30% after the first addition of the oxidant, 32% after the second addition, 34% after the third addition, and 39% after the fourth addition, with a degradation observed at the end of the process of 80%, significantly higher than that presented by the system in which the oxidant was added in a single time. In this system, TON [turnover number, defined as the ratio of amount of substance (mol) between the reacted dye and MnP] was ∼6, and TOF (turnover frequency) was ∼2 h^–1^.

Following the degradation reactions of the MB in this system, the catalyst was recovered by vacuum filtration in a Sintered Plate Funnel no. 4, thoroughly washed with distilled water, ethanol, methanol, dichloromethane, and ethyl ether, and then dried in the oven for 24 h at a temperature of 80 °C. This material was analyzed by ICP–MS, which verified a concentration of 11 μmol Mn per gram of Sil-Cl, denoting approximately 50% loss of MnP in the material.

Although there is a loss of MnP in the material, this result is promising, as the performance of the Sil-Cl@MnP catalyst may be remarkably improved by simply portioning the addition of oxidant to the system.

### 2.3 Phytotoxicity Tests

The products formed in degradation reactions of environmental pollutants may sometimes be more harmful than the initial pollutant itself.^51^ Given this possibility, this work evaluated the phytotoxicity of the mixture resulting from the catalytic process of MB degradation reactions, under the conditions in which the best degradation results were observed, i.e., at a Sil-Cl@MnP:MB:PhI(OAc)_2_ molar ratio of 1:23.5:188. As a model for phytotoxicity tests, the germination rate and root growth of seeds of the *Lactuca sativa* species were used (Table 2) as proposed by Young et al. (2012).^52^*L. sativa* seeds are successfully used to determine the phytotoxicity of varied pollutants that are released into the soil or bodies of water, such as industrial dyes,^53^ pesticides,^54^ and medicines.^55^ Among the advantages of this methodology are simplicity, repeatability, speed in obtaining results, and low cost, as it does not require sophisticated materials or equipment.^54^

**Table 2:** Phytotoxicity Assays with Lettuce Seeds (*Lactuca sativa*)

concentration	negative control^a^ (MB)	oxidant PhI(OAc)_2_^b^	MB + PhI(OAc)_2_	MB + PhI(OAc)_2_ + Sil-Cl@MnP	positive control^c^ (water)
Germination Rate (%)					
100%^d^	96,7^a^	0_b_	0^b^	0^b^	95^a^
30%	100,0^a^	91,7_a_	96,7^a^	100,0^a^	
10%	97,5^a^	86,5_a_	95,0^a^	92,5^a^	
Root Growth (cm)					
100%	2,61_b_	0_f_	0_f_	0_f_	2,65_b_
30%	2,83_a_	0,74_e_	1,57_d_	0,76_e_	
10%	2,84_a_	2,6_b_	2,47_b_	2,22_c_	

a Negative control MB: methylene blue aqueous solution.

b Iodobenzene diacetate.

c Positive control: distilled water.

d Refers to the concentration of 1 × 10^–3^ mol L^–1^; means followed by different letters indicate that there are statistical differences according to the Tukey test, with significance of 95%.

For the negative control (Table 2), only the aqueous solution of MB at the concentration of 1 × 10^–3^ mol L^–1^ was used, whereas for the positive control, only distilled water was used. Controls were also carried out with an aqueous solution of the oxidant (8 × 10^–3^ mol L^–1^) and with MB + oxidant in the same ratio as the degradation reaction was carried out. In general, there was no statistically significant influence (*p* > 0.05) on the germination rate when comparing the positive and negative controls, even in systems without dilution, denoting that MB was not toxic for the germination process of the *L. sativa* seeds. Conversely, when solutions containing PhI(OAc)_2_ were used without dilution, seed germination was not observed. However, when evaluating the seed germination rate with diluted oxidant solutions (30 and 10%), an increase in germination rates was observed, similar to the positive control (distilled water).

**Figure 10:**
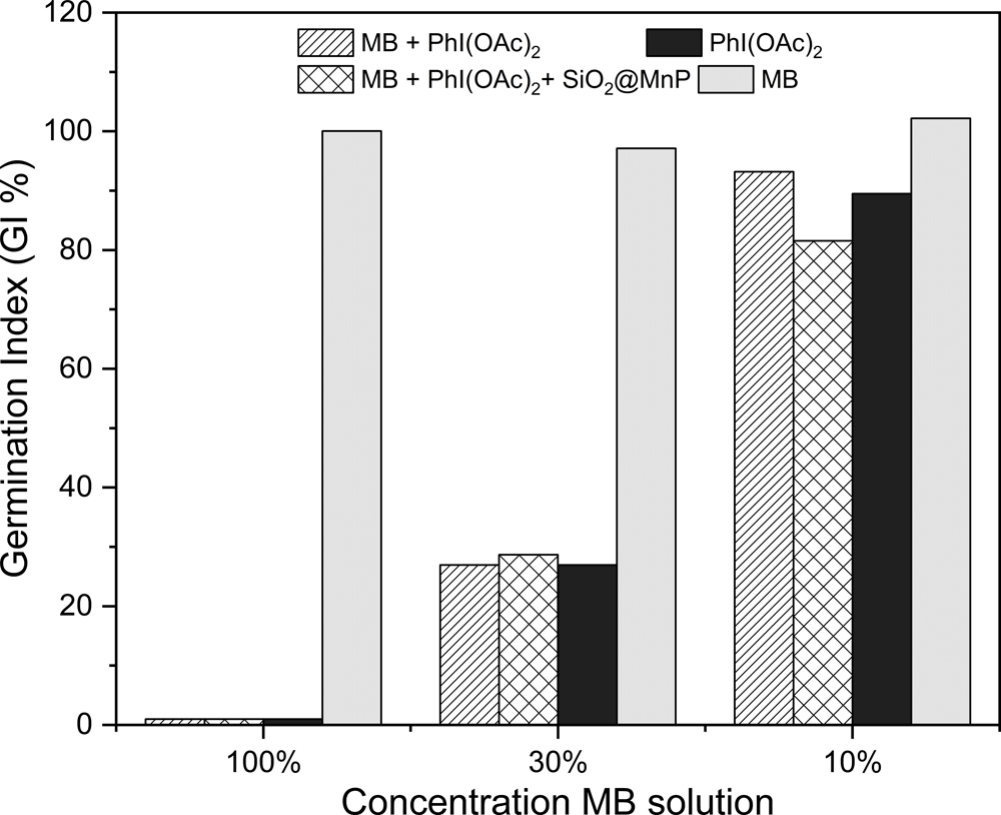
Effect of type of treatment on the germination index percentage (GI %) of *Lactuca sativa* seeds.

**Figure 11:**
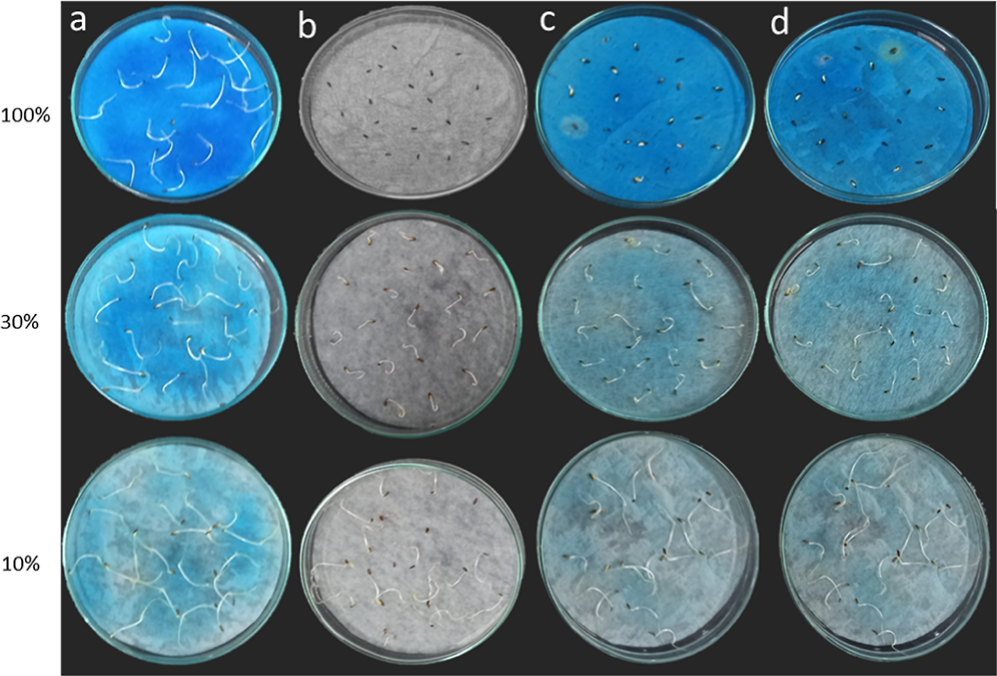
Root growth of samples: (a) MB. (b) Phl(OAc)_2_. (c) MB + PhI(OAc)_2_. (d) MB + PhI(OAc)_2_ + Sil-Cl@MnP at concentrations of 100, 30, and 10%.

No significant differences were observed in root growth in the systems using distilled water (positive control) and an undiluted MB solution (negative control). Controls containing the aqueous solution of PhI(OAc)_2_ without dilution showed no germination nor root growth. PhI(OAc)_2_ dilution was fundamental for root growth, and the more diluted the system (concentration of 10%), the closer to the result obtained with the negative control (no statistically significant differences, *p* > 0.05). In general, it was possible to infer that despite PhI(OAc)_2_ being toxic at high concentrations impairing the germination rate and root growth, a dilution of PhI(OAc)_2_ to 10% led to full recovery of the germination process and root growth rate, with no statistically significant difference from those of distilled water. According to Amado-Piña et al. (2022),^56^ germination indices (GI %) close to zero are considered high toxicity, below 50% are considered moderate toxicity, and close to 100% low toxicity, in relation to the control average. At a PhI(OAc)_2_ concentration of 30%, the GI averages were 27.5% for all treatments (including those with a catalyst), which can be considered moderate toxicity. On the other hand, at a PhI(OAc)_2_ concentration of 10% the GI values were 89.5% for oxidant PhI(OAc)_2_, 93.2% for the MB + PhI(OAc)_2_ combination, and 81.55% for MB + PhI(OAc)_2_ + Sil-Cl@MnP, presenting, this way, very low toxicity (Figures 10 and 11).

## 3 Conclusions

The search for efficient catalytic systems for the degradation of pollutants led to the development of a heterogenized catalyst through a covalent bond between the aminophenyl group of a Mn(III) porphyrin and the propyl substituent of functionalized chloropropyl silica gel. This catalyst showed high catalytic activity when used in MB degradation reactions using iodobenzene diacetate as an oxidant. Furthermore, after successive additions of oxidant, the catalyst maintained high performance, denoting high stability and effectiveness in the catalytic process carried out. It was also found that increasing the concentration of the oxidant led to a significant increase in the degradation of the dye. Through phytotoxicity tests, it was possible to verify toxicity in systems with PhI(OAc)_2_, however, in diluted systems the results were close to the positive control (distilled water only). Although the catalytic system presents high phytotoxicity without dilution, when diluted, it did not present phytotoxicity, highlighting the possibility of using Sil-Cl@MnP + PhI(OAc)_2_ for the degradation of MB. In this way, the Sil-Cl@MnP material has the potential to be further studied in environmental decontamination processes, for the degradation of various pollutants, such as other dyes, drugs, and pesticides, among others.

## 4 Experimental Section

### 4.1 Materials and Methods

#### 4.1.1 Reagents

The free base porphyrin *trans*-H_2_DAPDPP was obtained as a byproduct of the synthesis of 5-(4-aminophenyl)-10,15,20-trisphenylporphyrin (H_2_APTPP).^12^ Its characterization was reported in a work by our research group.^28^ Functionalized silica gel was originated from the work developed by Pinto and collaborators.^32^ The following reagents were used without prior purification: MB (C_16_H_8_ClN_3_S·*x*H_2_O; Fluka), reactive red 120 (C_44_H_24_Cl_2_N_14_NaO_20_S_6_; Sigma-Aldrich), iodobenzene diacetate (Sigma-Aldrich), and triethylamine [N(C_2_H_5_)_3_] (Sigma-Aldrich). All of the other reagents and solvents were of analytical grade and were used without further purification, unless stated otherwise.

#### 4.1.2 Equipment

UV–VIS spectra (190–1100 nm) were recorded on a Global Trade model GTA-97 spectrophotometer. Infrared (IR) spectra were registered on a PerkinElmer spectrometer model BXFTIR (4000 × 400 cm^–1^); the samples were prepared in KBr pellets. Room-temperature (25 °C) ^1^H NMR spectra were obtained in CDCl_3_ on a Bruker DPX-200 Advance spectrometer operating at 200 MHz; tetramethylsilane (TMS) was the internal standard. The ESI-MS analyses were conducted on an LCQFleet (Thermo-Scientific, San Jose, CA, USA) mass spectrometer equipped with electrospray ionization source operating in the positive ion mode and the analysis mode Time-Of-Flight (TOF); CH_3_OH was used as the solvent. UV–vis spectra with diffuse reflectance were recorded on a Thermo Scientific Evolution 600 spectrometer (200–900 nm) using BaSO_4_ as the reference. SEM analysis was performed on a JEOL JSM-6610LV/TMP scanning electron microscope with an X-ray spectrometer. The analyses were conducted under an accelerating voltage of 15 kV and low vacuum (1 mPa). Prior to analysis, the investigated material was coated with gold in order to improve the resolution of the images obtained. An inductively coupled plasma mass spectrometer 7700 (Agilent Technology, Tokyo, Japan) was used to determine Mn according to Costa et al. (2023).^57^

### 4.2 Manganese Porphyrin

17.7 mg (0.0274 mmol) of 5,15-diaminophenyl-10,20-diphenylporphyrin was dissolved (*trans*-H_2_DAPDPP) in 5 mL of chloroform in a round-bottom flask. Then, 57.1 mg (2.88 mmol) of manganese(II) chloride tetrahydrate, dissolved in 5 mL of methanol, was added to the flask containing the porphyrin. This system was kept at reflux and under magnetic stirring for approximately 15 h. MnP purification was initially carried out through liquid–liquid extraction. For this, the solvent was eliminated and the reaction medium was dissolved in CHCl_3_ and water was added to the system, with the MnP collected in the organic phase. Purification was then carried out by column chromatography, using silica (Sigma-Aldrich, 60 Å, 130–270 mesh) as the stationary phase and the solvent mixture CH_2_Cl_2_/CH_3_OH (5:1) as the eluent. The solvent was eliminated and the metalloporphyrin was stored and kept in a desiccator with silica gel.

Yield: 17.7 mg; 0.0241 mmol; 88%.

UV–vis in CHCl_3_, λ_max_ (nm) (log ε): 382 (4.30); 482 (4.64); 587 (3.58); 625 (3.74). FTIR (cm^–1^) in tablets KBr: (1620) δ NH_2_, (1294) δ of the porphyrin skeleton, (1010) δ Mn–N. (IT-TOF) Theoretical value [C_44_H_30_N_6_Mn]^+^, *m*/*z* 697.1912. Obtained value: *m*/*z* 697.2020.

### 4.3 Immobilization of MnP to Sil-Cl

50 mL of a 1.2 × 10^–3^ mol L^–1^ solution of *trans*-[Mn^III^(DAPDPP)Cl] in CH_2_Cl_2_ were placed in a round-bottom flask, along with 1000 g of chloropropyl silica gel and 1.0 mL of triethylamine. This system was kept under reflux and magnetic stirring for approximately 24 h. Then, the silica was washed in a *N*° 4 sintered plate funnel with different solvents in this order (hexane, dichloromethane, chloroform, ethyl acetate, acetone, ethanol, methanol, distilled water, and ether), until the characteristic green color of MnP was no longer observed in the washing solvents. Finally, the dark green material was left in the oven for 24 h at 100 °C to dry. The amount of Mn porphyrin immobilized onto Sil-Cl (loading) was determined directly by ICP–MS determining the amount of manganese in Sil-Cl and Sil-Cl@MnP. For this analysis, the samples were placed in concentrated HNO_3_ overnight. They were then heated in a water bath (90–100 °C) for 30 min. The digests were measured to 50.0 mL, centrifuged and diluted 10 times, and then analyzed by ICP–MS. The surface charge of the material (Sil-Cl@MnP) was measured in a folded capillary cell by zeta potential measurements performed in aqueous solutions of the particles (pH within the range 5.9–6.1) using Zetasizer Nano ZS equipment from Malvern Instruments (Malvern, UK).

### 4.4 Dye Degradation Reactions

All dye degradation reactions (MB or reactive red 120 as substrates) were carried out in 10 mL penicillin vials. The reactions were carried out under magnetic stirring, at 25 ± 2 °C in the absence of light in an aerobic environment, using distilled water as a solvent and PhI(OAc)_2_ or H_2_O_2_ as oxidant. The penicillin vials containing 7.4 mg of Sil-Cl@MnP (1.70 × 10^–7^ mol of MnP) and 4.0 mL of an aqueous dye solution (1 × 10^–3^ mol L^–1^) were kept under magnetic stirring for 2 h to ensure adsorption balance between the dye and the catalyst surface. Then, the oxidant (PhI(OAc)_2_ or H_2_O_2_) was added, starting the catalytic reaction time (time zero). Different molar ratio dye:oxidant was evaluated. The percentage of dye degradation was determined by UV–vis absorption spectroscopy, using eq 1, for this, 30 μL of the reaction solution was added to a cuvette containing 2.0 mL of distilled water.
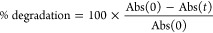
1

The absorbance of the dye at time zero [Abs(0)] refers to the absorbance immediately before the addition of the oxidant (after the adsorption process), and the absorbance of the dye at time *t* [Abs(*t*)] refers to the absorbance at a particular sampling time stipulated for each system. The efficiency of the catalyst was also analyzed by making successive additions of the oxidant, with the oxidant being added to the system every 2 h of reaction. All experiments were carried out at least in duplicate and expressed the averages of these values. Control reactions were carried out (a) in the absence of the catalyst and oxidant, (b) in the absence of the catalyst, and (c) in the absence of the oxidant.

### 4.5 Phytotoxicity Tests

To carry out the phytotoxicity tests, an adaptation to the protocol described by Young et al. (2012)^52^ was carried out. In Petri dishes, 20 lettuce seeds were placed on paper disks for germination (Whatman) saturated with 2 mL of the reaction supernatant sample at different concentrations (without dilution, 10 and 30% v/v). The plates were sealed with film paper and plastic bags to avoid the loss of humidity and incubated for 5 days at a temperature of (22 ± 2) °C in a dark place. Distilled water was used as a positive control, and as negative controls the original aqueous solution of the dye was used for degradation (without dilution, 1 × 10^–3^ mol L^–1^) and diluted to 10 and 30% (v/v). All assays were performed in triplicate. After the incubation period, the effects were quantified: on relative germination (RG %), relative root growth (RRG %), and germination index (GI %), using the following equations, according to the work described by Tam and Tiquia^58^
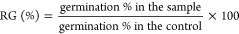
2
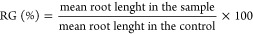
3
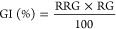
4

Statistical analyzes were performed using the Tukey test at 5% probability using the statistical program R.^59^

## Supporting Information Available

Mass spectrum (ESI-MS) and IR spectra for MnP and IR spectra for Sil-Cl@MnP (PDF)
