# Supplementary material for: Degradation of Dyes Catalyzed by Aminophenyl-Substituted Mn-Porphyrin Immobilized on Chloropropyl Silica Gel and Evaluation of Phytotoxicity
Source: ACS Omega. 2024 Jun 25;9(27):29516–28. doi: 10.1021/acsomega.4c02132 (PMC11238201; doi:10.1021/acsomega.4c02132)
Supplement: Supplementary file 1 — ao4c02132_si_001.pdf [file ao4c02132_si_001.pdf]

Supporting Information

Degradation of dyes catalyzed by aminophenyl-substituted Mn-porphyrin immobilized on chloropropyl silica gel and evaluation of phytotoxicity

Igor Muniz de Oliveira[1], João Victor Docílio Pereira[2], Everton Carlos da Silva Pereira[2], Micaelle Silva de Souza[3], Márcia Luciana Cazetta[2], Claudiano Carneiro da Cruz Neto[3], Victor Mancir da Silva Santana[4], Victor Hugo Araújo Pinto[5], Júlio Santos Rebouças[5], Dayse Carvalho da Silva Martins[6], Gilson DeFreitas-Silva[6], Denilson Santos Costa[7], Vinicius Santos da Silva[2*].

*e-mail: vinicius.sdasilva@ufrb.edu.br

[1]Centro de Formação de Professores, Universidade Federal do Recôncavo da Bahia, 45300-000. Amargosa, BA, Brazil.

[2] Centro de Ciências Exatas e Tecnológicas – Universidade Federal do Recôncavo da Bahia, 44380-000. Cruz das Almas, BA, Brazil.

[3] Centro de Ciências Agrárias Ambientais e Biológicas – Universidade Federal do Recôncavo da Bahia, 44380-000. Cruz das Almas, BA, Brazil.

[4] Instituto de Física, Universidade Federal da Bahia, 40210-340 Salvador, BA, Brazil.

[5] Departamento de Química, CCEN, Universidade Federal da Paraíba, João Pessoa, PB, Brazil

[6] Departamento de Química, Instituto de Ciências Exatas, Universidade Federal de Minas Gerais, 31270-901. Belo Horizonte, MG, Brazil.

[7] Instituto de Química, Universidade Federal da Bahia, 40170-115. Salvador, BA, Brazil.

***Corresponding author:**

Prof. Vinicius Santos da Silva (da Silva, V.S.)

Centro de Ciências Exatas e Tecnológicas – Universidade Federal do Recôncavo da Bahia, Cruz das Almas, BA, 44380-000, BRAZIL

Tel.: +55 75 3621 4314;

E-mail address: vinicius.sdasilva@ufrb.edu.br

1. Manganese porphyrin (MnP)

Mass spectral analysis (ESI-MS) confirmed metal insertion into the second-generation *trans*-H_2DAPDPP: a peak centered at m/z 697.2020 emerged in the spectrum recorded in the positive mode, which corresponded to *cis*-[Mn^{III}DAPDPP]$^+$ after loss of the chloride counterion, Figure S1.

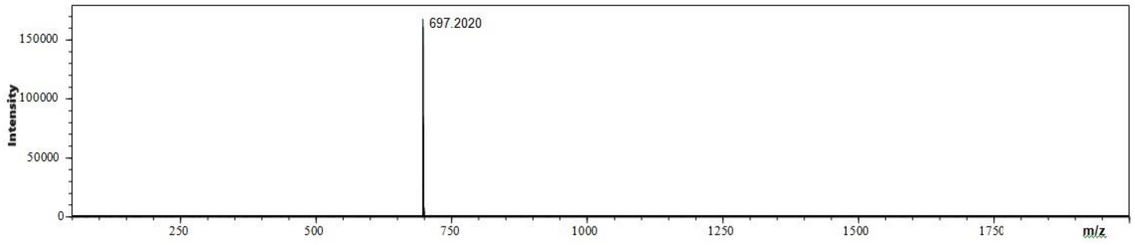

Figure S1. Mass spectrum of *trans*-[Mn^{III}(DAPDPP)Cl]. Analysis conducted in CH_3OH with the ESI-MS operating in the positive mode.

By analyzing the vibration spectrum in the infrared region (Figure S2), there was a band at 1620 cm^{-1} that can be associated with the deformation of the -NH_2, a deformation at 1294 cm^{-1} referring to the displacement associated with the porphyrin skeleton and the band at 1010 cm^{-1}, which can be related to the displacement of the Mn-N bond [1].

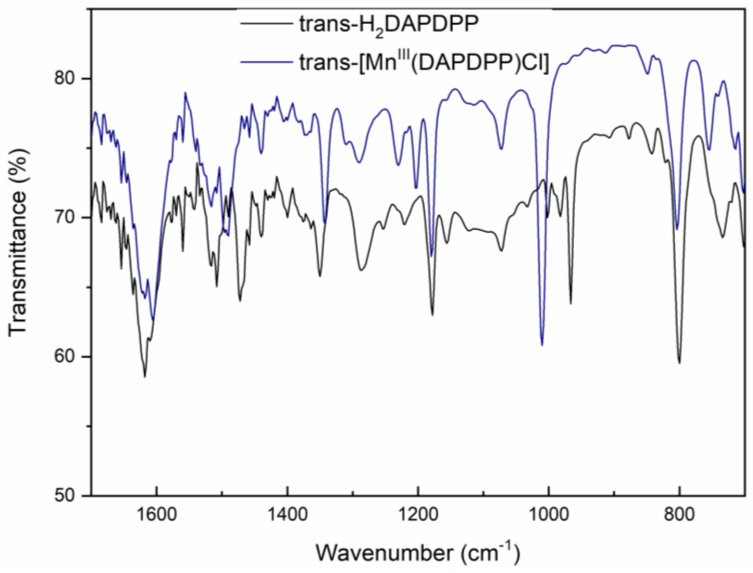

Figure S2. Infrared absorption spectra of *trans*-H_2DAPDPP and *trans*-[Mn^{III}(DAPDPP)Cl] in KBr pellets.

The catalytic material SilCl@MnP was characterized by FTIR, (Figure S3). In this spectrum it was observed the vibrations modes at 3465 cm^{-1} (O-H stretching) and at 1630 cm^{-1} (O-H bending) assigned to the silanol groups. The bands corresponding to the siloxanes groups are located at 1100 cm^{-1} (asymmetric stretching of Si-O-Si), 800 cm^{-1} (symmetric stretching of Si-O-Si), and 468 cm^{-1} (bending of Si-O-Si). The weak band observed at 2960 cm^{-1} may be associated with the presence of the carbon chain of chloropropyl groups [2].

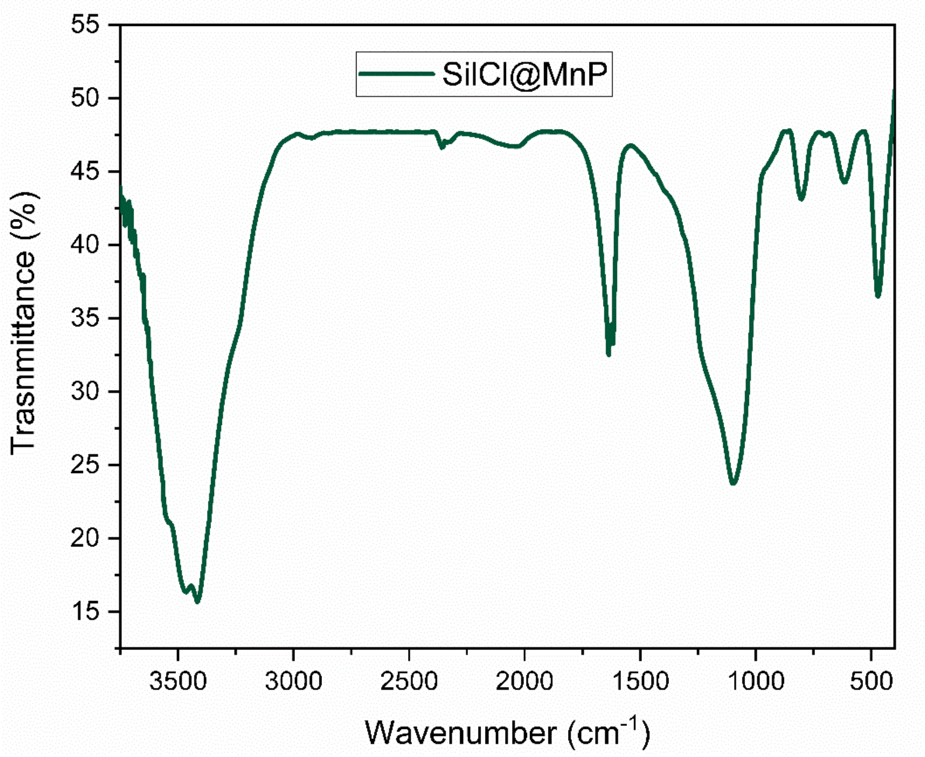

Figure S3. Infrared absorption spectra of Sil-Cl@MnP in KBr pellets.

Reference

[1] M. Gouterman, in: D. Dolphin (Ed.), The Porphyrins, Academic Press, Ney York, 1978, pp. 1 -165.

[2] Pinto, V. H. A.; Falcao, N.; Mariz-Silva, B.; Fonseca, M. G.; Reboucas, J. S. Robust Mn(III) N-pyridylporphyrin-based biomimetic catalysts for hydrocarbon oxidations: heterogenization on non-functionalized silica gel versus chloropropyl-functionalized silica gel. *Dalton Trans.* **2020**, *49* (45), 16404-16418. DOI: 10.1039/d0dt01383h.
